# Supplementation with spinach-derived thylakoid augments the benefits of high intensity training on adipokines, insulin resistance and lipid profiles in males with obesity

**DOI:** 10.3389/fendo.2023.1141796

**Published:** 2023-07-28

**Authors:** Ayoub Saeidi, Mohammad Amin Saei, Behnam Mohammadi, Hamid Reza Akbarzadeh Zarei, Morvarid Vafaei, Amir Shayan Mohammadi, Masoumeh Barati, Mona Montazer, Omid Razi, Maisa Hamed Al Kiyumi, Ismail Laher, Mandana Gholami, Katja Weiss, Beat Knechtle, Hassane Zouhal

**Affiliations:** ^1^ Department of Physical Education and Sport Sciences, Faculty of Humanities and Social Sciences, University of Kurdistan, Sanandaj, Iran; ^2^ Department of Sports Sciences, Faculty of Humanities, Tarbiat Modares University, Tehran, Iran; ^3^ Department of Exercise Physiology, Central Tehran Branch, Islamic Azad University, Tehran, Iran; ^4^ Department of Physical Education and Sport Sciences, Science and Research Branch, Islamic Azad University, Tehran, Iran; ^5^ Department of Exercise Physiology, Faculty of Physical Education and Sports Science, Razi University, Kermanshah, Iran; ^6^ Department of Family Medicine and Public Health, Sultan Qaboos University, Sultan Qaboos University Hospital, Muscat, Oman; ^7^ Department of 87 Anesthesiology, Pharmacology and Therapeutics, The University of British Columbia, Vancouver, BC, Canada; ^8^ Institute of Primary Care, University of Zurich, Zurich, Switzerland; ^9^ Medbase St. Gallen Am Vadianplatz, St. Gallen, Switzerland; ^10^ Univ Rennes, M2S (Laboratoire Mouvement, Sport, Santé), Rennes, France; ^11^ Institut International des Sciences du Sport (2I2S), Irodouer, France

**Keywords:** functional training, adipokines, insulin resistance, obese men, supplementation

## Abstract

**Introduction:**

This study investigated the effects of 12 weeks of high-intensity functional training (HIFT) combined with spinach-derived thylakoid supplementation on some selected Adipokines and insulin resistance in males with obesity.

**Method:**

Sixty-eight participants (mean age: 27.6 ± 8.4 yrs.; mean height: 168.4 ± 2.6 cm; mean weight: 95.7 ± 3.8 kg, mean BMI: 32.6 ± 2.6 kg/m^2^) were randomly divided into four groups of 17 per group: Control group (CG), Supplement group (SG), Training group (TG), and Training + supplement group (TSG). Following baseline measurements, the two training groups (TG and TSG) started the 12 weeks of exercise training program (3 sessions per week). A total of 36 sessions lasting up to 60 min were included in the HIFT program using the CrossFit program. The eligible participants received 5 g/day of thylakoid-rich spinach extract or matching placebo as 5 g/day of raw corn starch (one sachet, 30 min before lunch) for 12 weeks. Baseline assessments were obtained 48 hours before the start of the training protocols and 48 hours after the last training session in all groups.

**Results:**

There were significant interactions (p<0.001 for all) between exercise and time for adiponectin (ES:0.48), leptin (ES:0.46), resistin (ES:0.3), omentin (ES:0.65), vaspin (ES:0.46), visfatin (ES:0.62), apelin (ES:0.42), RBP4 (ES:0.63), chemrin (0.36) and semaphorin3c (ES: 0.5). Plasma levels of semaphorin3c were significantly correlated (p<0.05) with body weight (r= 0.57), BMI (r= 0.43), FFM (r= -0.612), FAT (r= 0.768), VO_2peak_ (r=-0.53), insulin (r= 0.756), glucose (r= 0.623), and HOMA-IR (r= 0.727). There were also significant group differences in insulin (ES: 0.77), glucose (ES: 0.21), and HOM-IR (ES: 0.44) (p<0.05).

**Discussion:**

Our findings indicate that 12 weeks of HIFT supplemented with spinach-derived thylakoid reduced levels of leptin, resistin, vaspin, visfatin, apelin, RBP4, chemrin, semaphorin3c and insulin resistance while increasing adiponectin and omentin levels in men with obesity.

## 1 Introduction

Obesity is a chronic condition with increased global prevalence (1). Obese individuals are at increased risk for hyperlipidemia, type 2 diabetes mellitus (T2DM), cardiovascular disease (CVD), stroke, hypertension (HTN), some cancers, sleep apnea, and nonalcoholic fatty liver disease (NAFLD) (2). Adipose tissue dysfunction plays an important role in the development of obesity associated disorders (3), and is related to dysregulated (upregulation of inflammatory and downregulation of anti-inflammatory adipokines) release of adipokines (4) such as apelin, chemerin, visfatin, resistin, adiponectin, leptin, retinol binding protein (RBP4) (5), and semaphorin 3C (6). Adipokines are promising pharmacological treatment targets for obesity and metabolic disorders as they regulate appetite and satiety, energy expenditure, blood pressure, endothelial function, insulin sensitivity, adipogenesis, fat distribution and insulin secretion (7). Exercise training, calorie restriction or their combination are effective strategies in preventing or treating obesity (8). Regular physical exercise is associated with improved body composition, including decreased body weight and abdominal adiposity (9), improvements in lipid profiles, including decreasing cholesterol and LDL, increased HDL levels (10), and improved glucose homeostasis and insulin sensitivity (11). Regular exercise also reduces chronic inflammation (12), improves endothelial function and reduces blood pressure in prehypertensive or hypertensive patients (13). Exercise decreases inflammation related adipokine expression (14), and attenuates the dysregulated expression of adipokines and oxidative stress in white adipose tissue (15). Exercise lowers circulating levels of proinflammatory adipokines and causes simultaneous increases in anti-inflammatory adipokines including adiponectin (16).

High-intensity functional training (HIFT) is a newly described exercise training program that emphasizes functional, multi-joint movements, that can be prescribed for individuals with all fitness levels, and causes greater muscle recruitment compared to traditional exercise training (17). HIFT is associated with improvements in body composition and glucose control variables in overweight and obese individuals (18), while also improving β-cell function in patients with T2DM (19). These findings suggest that HIFT can decrease body fat of young overweight and obese individuals, while being more effective than endurance training in improving neuromuscular performance (20). Some studies suggest that in addition to exercise training, thylakoids, membrane proteins extracted from green leaves such as spinach, can reduce weight in humans by inducing satiety, decrease appetite and possibly food intake through homeostatic and non-homeostatic pathways (21). In addition, thylakoid-rich spinach extracts combined with a weight loss intervention (low-calorie diet) affected circulating levels of adipokines, enhanced serum levels of omentin-1 and decreased fat mass, abdominal obesity and serum chemerin in obese women (22).

Despite the positive effects of different types of exercise training including HIFT, and the benefits of herbal supplements such as thylakoid-rich spinach extracts in managing obesity and combating its related metabolic disorders, there is limited information on HIFT alone or in combination with spinach-derived thylakoid ingestion on obesity and adipose tissue secreted adipokines. We hypothesized that HIFT supplemented with ingestion of spinach-derived thylakoid can amplify the effects of HIFT on obesity and circulating adipokines. This study aimed investigated the effects of 12 weeks of HIFT with or without spinach-derived thylakoid supplementation on the levels selected adipokines, insulin resistance and lipid profile in obese men.

## 2 Materials and methods

The study had an initial recruitment of 100 male volunteers, of which 32 were ineligible and leaving 68 participants in the study (mean age: 27.6 ± 8.4 yrs.; mean height: 168.4 ± 2.6 cm; mean weight: 95.7 ± 3.8 kg, mean BMI: 32.6 ± 2.6 kg/m^2^). Inclusion criteria included: BMI > 30 kg/m^2^, not involved in regular physical activity during the last six months, no evidence of cardiovascular, metabolic, and endocrine diseases, and not consuming alcohol. Participants with joint disorders, physical disabilities, and those taking prescribed medications and any supplements that could affect muscle and adipose tissue metabolism were excluded from the study. All participants completed a physical examination performed by a physician and clinical exercise physiologist on the first visit and provided a written consent form and Physical Activity Readiness Questionnaire (PAR-Q) (23). The Research and Ethics Committee of the Islamic Azad University (Damghan Branch) approved all procedures of this study (Ethics code: IR.IAU.DAMGHAN.REC.1401.034). The research was also registered at the Iranian Registry of Clinical Trials (https://irct.ir/trial/69048) with code IRCTID: IRCT20151228025732N77. All procedures were performed according to the latest revision of the Declaration of Helsinki.

### 2.1 Experimental design

The participants were familiarized with all study procedures one week prior to the start of the training programs. Height, weight, and body composition were measured for each of the participants, who were then randomly assigned into one of the four equally sized groups (17 per each): Control group (CG), Supplement group (SG), Training group (TG), and Training + supplement group (TSG). During the study, 8 participants from different groups withdrew from the study for medical reasons, employment related difficulties or lack of interest in continued participation, leaving 15 participants per group. Each group received instructions on performing the training protocols during the third session when body composition variables and VO_2peak_ were also measured. Following baseline measurements, the two training groups (TG and TSG) started the 12 weeks of training programs (3 sessions per week). Participants in the control group were instructed to maintain their current lifestyles until the end of the study. Study measurements were collected at the same time of day (within ~1 hour) and under the same environmental conditions (~20°C and ~55% humidity). Baseline assessments were obtained 48 hours before the start of the training protocols and again 48 hours after the last session in all groups. Participants in the training protocols were asked to follow the same diet for 48 hours before the baseline assessment and also before the final measurements.

### 2.2 Body composition and cardio-respiratory fitness assessments

Body weights and heights were measured using a calibrated scale (Seca, Germany) and stadiometer (Seca, Germany), respectively, and were used to calculate body mass index (BMI) (kg/m^2^). Levels of fat-free mass (FFM) and fat mass (FM) were measured by a bio-impedance analyzer (Medigate Company Inc., Dan-dong Gunpo, Korea), and VO_2peak_ was determined using a modified Bruce protocol (in a temperature-controlled room maintained at 21–23°C) as reported in previous studies of overweight and obese populations (24, 25), using an electrically motorized treadmill (H/P/Cosmos, Pulsar med 3p- Sports and Medical, Nussdorf-Traunstein Germany). The physiological criteria used to determine VO_2_peak were based on the American College of Sports Medicine (ACSM) guidelines based on participants reporting that they were physically exhausted and reached their maximal effort (according to the Borg scale), or if the supervisor recognized the participants had severe dyspnea, dizziness and other limiting symptoms based on the guidelines for cardiopulmonary exercise test (CPET) tests of the ACSM and American Heart Association (AHA) (26, 27), with a plateau in VO_2_ and respiratory exchange ratio (RER) ≥1.10. Blood pressure was measured using an electronic sphygmomanometer (Kenz BPM AM 300P CE, Japan), and heart rate was monitored with a Polar V800 heart monitor (Finland) throughout the tests. Gas analysis was performed using a gas analyzer system (Metalyzer 3B analyzer, Cortex: biophysik, GMbH, Germany), which was calibrated before each test.

### 2.3 Preparation of spinach thylakoids and placebo

Fresh baby spinach leaves (Spinacia oleracea) were used to prepare of thylakoid membranes according to previously registered protocols (28–30). Fresh spinach was collected from Tabriz, East Azerbaijan Province, Iran during the spring season of 2020. The *Spinacia oleracea* L. (belonging to the Oleracea family) samples we collected has a herbarium number of TBZ-fph-1898. The thylakoid supplement used in this investigation was prepared based on the method described by Emerk et al. (30). The stems and veins were removed from fresh spinach leaves, and then soaked in cold water and cleaned. Spinach leaves (1000 g) were homogenized with 1250 ml water in a blender and filtered through four layers of Monodur polyester mesh (20 μm), and the resulting filtrate was diluted 10 times with distilled water, and the pH adjusted to 4.7 with hydrochloric acid (HCl). The isoelectric point of thylakoids (pH 4.7) allows for maximum precipitation. The thylakoids flocculated, with a green precipitate and a clear, slightly yellowish supernatant after 4 h standing in the cold (− 4°C). The supernatant was removed, and the green precipitate (thylakoids) was collected from the filtrate at pH 4.7 and washed in water by repeated centrifugation; the precipitation was then repeated again at pH 4.7. The washed thylakoids were collected, the pH adjusted to 7.0 and the final sediments were freeze-dried to obtain a green thylakoid powder. Placebo consisted of corn starch, which was colored with edible green color that matched the thylakoid powder and that was flavored with kiwi fruit essence. Corn starch, a substance without any therapeutic effects, is a white, tasteless, safe, non-toxic, non-irritant, and non-allergenic odorless powder that is commonly used in the food and pharmaceutical industries.

Powder samples were packed in identical sachets with each sachet containing 5 g of thylakoid or 5 g of cornstarch powder. The contents of the sachets were dissolved in a glass of water and consumed by participants 30 min before lunch. Packages were coded and distributed monthly by a third person who was not involved in any other aspects of the study. A supplement consumption chart, which was provided to remind participants to consume their daily supplements, was returned at each visit to ensure compliance. Participants also received daily text messages and weekly phone call reminders to consume the supplements. The participants were asked to return the remaining sachets at each visit to allow monitoring of; compliance. Adherence was deemed acceptable when ≥80% of the supplements were consumed.

### 2.4 Training protocols

Participants completed 36 sessions in the HIFT program that lasted up to 60 min per session. The CrossFit program was used and HIFT sessions were led by a CrossFit trainer where the first two sessions were structured as an introduction to common movements used in HIFT (e.g., squats, deadlift, press, jerks, barbell, dumbbell, and medicine ball cleans, pull-ups, kettlebell swings, among others) with no additional workouts on days 1 and 2. Starting on day 3, each HIFT class consisted of 10–15 min of stretching and warm up, 10–20 min of instruction and practicing techniques and movements, and 5–30 min for the workout of the day that was performed at vigorous intensity and relative individual levels of ability and fitness. Workout modalities included aerobic (e.g., running, jumping rope), body weight (e.g., pull-ups, squats), and weightlifting (e.g., front squats, kettlebell swings) exercises that were constantly varied using the CrossFit training template (31) in single, couplet, or triplet modalities that were completed for time, repetitions, or weight. All weights and movements were individually prescribed and recorded for each HIFT participant (32). The times to complete the workout of the day, rounds and repetitions completed on the workout of the day, weights used, and any modifications needed from the programmed workout were recorded for each participant. Average times and total average workout of the day time per week were calculated for the entire HIFT group.

### 2.5 Nutrient intake and dietary analysis

Three-day food records (two weekdays and one weekend day) were obtained before and after the study to assess changes in habitual dietary intake over time (33). Each food item was individually entered into Diet Analysis Plus version 10 (Cengage, Boston, MA, USA), and total energy consumption and the amount of energy derived from proteins, fats, and carbohydrates were determined *(* **Table 1** *)*.

**Table 1:** Nutritional intake in the four study groups.

	CG			SG			TG	TSG	
	Pre- study	Post-study	Pre- study	Post-study	Pre-study	Post-study		Pre- study	Post-study
Energy (kcal/d)	2255 ± 67	2261 ± 86	2274 ± 111	2132 ± 150	2253 ± 127	2161 ± 167		2271 ± 177	2110 ± 186
Carbohydrates (g/d)	280 ± 12.4	282 ± 19.3	276.4 ± 77.1	260 ± 67.5	283 ± 48.6	264 ± 19.2		288 ± 18.6	261 ± 19.1
Fat (g/d)	81.2 ± 10.0	80 ± 8.8	85.5 ± 11.7	74 ± 13.2	80.4 ± 14.4	72.1 ± 13.2		79.8 ± 10.87	70.2 ± 15.3
Protein (g/d)	103 ± 11.0	105 ± 13.3	100 ± 14.5	94 ± 11.6	102 ± 17.8	92 ± 12.7		103 ± 15.5	89 ± 14.5

CG, Control group; SG, Supplement group; TG, Training group; TSG, Training supplement group. *Indicates significant differences compared to pre-study values (p<0.05).

### 2.6 Blood markers

All tests were performed between 8-10 am under standard conditions of temperature and humidity. Fasting blood samples were taken from the right arm between 12 hours and 72 hours before the first exercise session and 72 hours after the last session. Blood samples were transferred to EDTA-containing tubes, centrifuged for 10 minutes at 3000 rpm, and stored at -70°C. Plasma glucose levels were measured with a colorimetric enzymatic kit (Parsazmun, Tehran, Iran) with a sensitivity of 5 mg/dl. Insulin resistance was assessed using the homeostasis model assessment of insulin resistance (HOMA) using the formula: HOMA-IR= 22.5 μmol/fasting plasma insulin X fasting plasma glucose. Plasma resistin was measured with an ELISA kit (Biovendor, Czech Republic, catalogue number RD191016100, sensitivity: 0.012 ng/ml, intra-CV = 5.9%, inter-CV = 7.6%). Plasma leptin was measured with an ELISA kit (Biovendor, Czech Republic, catalogue number: RD191001100, sensitivity: 0.2 ng/ml, intra-CV = 5.9%, inter-CV = 5.6). Plasma adiponectin was measured with an ELISA kit (Biovendor, Czech Republic, catalogue number: RD19502310, sensitivity: 26 ng/ml, intra-CV = 4.9%, inter-CV = 6.7%). Plasma visfatin was measured with an ELISA kit (Cusabio, China, catalogue number: CSB-E08940h, sensitivity: 0.156 ng/mL, intra-CV = <8%, inter-CV = <10%). Plasma vaspin was measured with an ELISA kit (Biovendor, Czech Republic, catalogue number: RD191097200R, sensitivity: 0.01 ng/ml, intra-CV = 7.6%, inter-CV = 7.7%). Plasma RBP-4 was measured with an ELISA kit (R&D Systems, USA, catalogue number: DRB400, sensitivity: 0.628 ng/mL, intra-CV = 7%, inter-CV = 8.6%). Plasma apelin was measured with an ELISA kit (Phoenix Pharmaceuticals, USA, catalogue number: EK-057-23, sensitivity: 0.07 ng/ml, intra-CV = <10%, inter-CV = <15%). Plasma omentin-1 was measured with an ELISA kit (Biovendor, Czech Republic, catalogue number: RD191100200R, sensitivity: 0.5 ng/ml, intra-CV = 3.7%, inter-CV = 4.6%). Plasma chemerin was determined using a commercially available ELISA kit (Biovendor, Czech, intra-assay coefficient of 5.1%). The plasma levels of semaphorin 3C (MBS037239, MBS2883689, MyBioSource, San Diego, USA) was measured with a commercially available ELISA kit. Insulin levels were measured with an ELISA kit (Demeditec, Germany, with a sensitivity of 1 ng/ml and between, within-coefficients of variation of 5.1% and 8.4% respectively. Glucose levels were measured with a colorimetric enzymatic kit (Parsazmun, Tehran, Iran, with a sensitivity of 5 mmol/l). Insulin resistance (IR) was calculated from the ratio of insulin to glucose (I0/G0) and the HOMA-IR index [HOMA-IR = fasting insulin (mU/L) × glucose (mmol/L)/22.5] (34).

### 2.7 Statistical analysis

*A priori* sample size calculation was conducted using G-Power 3.1.9.2 software. The rationale for the sample size was based on previous work on combined training which documented significant reductions in leptin levels in overweight and obese males (35). By utilizing the equation for effect size (ES) [(mean before-mean after combined aerobic and resistance training)/the pooled standard deviation], this study revealed an ES of 1[(5.4–3.6)/1.65]. In our study that was based on α = 0.05, a power (1- β) of 0.95, and an ES = 1 (highest approximate effect size), a total sample size of at least 20 participants (n = 5 per group) was needed for sufficient power to detect significant changes in leptin levels. However, since no previous study reported the effects of CrossFit on the adipokines we measured and the fact that COVID-19 might negatively affect training and supplementation compliance, we increased the sample size (n=17) to maintain the study power. Descriptive statistics (means ± standard deviation) were used to describe all data. The normality of the data was assessed by the Shapiro–Wilk test. A two-way ANOVA repeated measures test was used to determine groups X time interactions. One-way ANOVA and Fisher’s Least Significant Difference *post-hoc* tests were used for evaluation baseline data of four groups. When a significant difference was detected by ANOVA, mean differences were determined by pairwise comparisons. The associations between Semaphorin3c concentrations and other variables were measured using Pearson correlation tests. The sample size was calculated to detect a statistical difference between study variables with a 95% confidence interval (CI) and equal or greater than 80% of power value. Effect sizes (ES) were reported as partial eta-squared, and were considered trivial (< 0.2), small (0.2-0.6), moderate (0.6-1.2), large (1.2-2.0) and very large (2.0-4.0) (36). A p-value of <0.05 was used to indicate statistical significance. All data were analyzed using SPSS software (version 24).

## 3 Results

Measurements of nutritional intake variables are presented in **Table 1**, where there were no significant differences between the groups in energy, and intakes of carbohydrates, fat and protein (*p>0.05*). In addition, while baseline levels of energy, and consumption of carbohydrates, fat and protein were similar in all study groups (*p>0.05*), there were no significant pre-differences in post-test values for variables in all groups compared to pretest values (p>0.05).

Measurements of body composition and cardio-respiratory fitness variables are presented in **Table 2**, where there were significant differences between the groups in body weight (ES: 0.37), fat-free mass (FFM), (ES: 0.23), fat mass (ES: 0.42), insulin (ES: 0.77), glucose (ES: 0.21), and HOM-IR (ES: 0.44) (p<0.05), with no differences in BMI (p=0.11, ES: 0.14) or VO_2_ peak (p=0.27, ES: 0.09) between the groups. Moreover, group and time interactions for these variables were significant (p<0.05, ES range: 0.43- 0.96). In addition, while baseline levels of body weight, FFM, fat mass, insulin, glucose, and HOM-IR were similar in all the study groups (p>0.05), there were differences in post-test values for variables in all groups compared to pretest values (p<0.05). No changes were observed in the control group (p>0.05).

**Table 2:** Mean ( ± SD) and effect sizes of logical anthropomorphic levels in the all groups.

Variables	Group	Pre-training Mean (SD)	Post-training Mean (SD)	p- values (η2)		
				Time	G × T Interaction	Group
Weight (kg)	CG	94.33 (1.82)	93.55 (2.43)	<0.001 (0.63)	<0.001 (0.44)	<0.001 (0.37)
	SG	93.28 (2.61)	91.13 (2.12)			
	TG	92.78 (1.89)	89.19 (2.37)			
	TSG	94.13 (1.90)	87.25 (2.30)			
BMI (kg/m^2^)	CG	33.08 (1.34)	32.87 (1.44)	<0.001 (0.63)	<0.001 (0.45)	0.11 (0.14)
	SG	32.66 (1.37)	31.93 (.93)			
	TG	33.22 (1.07)	31.85 (1.19)			
	TSG	33.05 (.75)	30.68 (.95)			
VO2peak (mL·kg^-1^·min^-1^)	CG	27.27 (2.41)	26.90 (2.25)	<0.001 (0.62)	<0.001 (0.62)	0.27 (0.09)
	SG	27.09 (2.84)	27.54 (2.91(			
	TG	27.36 (2.37)	29.90 (1.92(			
	TSG	27.00 (2.04)	30.18 (2.22)			
FFM (kg)	CG	27.63 (1.20)	26.54 (2.25)	<0.001 (0.46)	<0.001 (0.43)	0.01 (0.23)
	SG	27.09 (1.81)	29.36 (.92)			
	TG	26.72 (1.27)	29.54 (1.5)			
	TSG	27.18 (1.77)	30.36 (1.2)			
FAT (%)	CG	30.09 (1.51)	30.83 (2.05)	<0.001 (0.54)	<0.001 (046)	<0.001 (0.42)
	SG	30.10 (1.59)	28.08 (.79)			
	TG	30.36 (1.50)	26.89 (.95)			
	TSG	31.13 (1.35)	26.62 (1.21)			
Insulin (ng/ml)	CG	18.81 (.67)	19.12 (.57)	<0.001 (0.84)	<0.001 (0.78)	<0.001 (0.77)
	SG	18.80 (.75)	17.60 (.51)			
	TG	18.83 (.40)	16.12 (.43)			
	TSG	19.12 (.49)	15.51 (.55)			
Glucose (mg/dl)	CG	96.44 (13.10)	90.73 (6.43)	<0.001 (0.8)	<0.001 (0.5)	0.02 (0.21)
	SG	98.99 (10.71)	84.77 (4.50)			
	TG	99.27 (5.72)	74.08 (5.43)			
	TSG	101.63 (7.13)	71.51 (7.71)			
HOMA-IR	CG	4.48 (.70)	4.27 (.30)	<0.001 (0.87)	<0.001 (0.7)	<0.001 (0.44)
	SG	4.58 (.46)	3.68 (.25)			
	TG	4.61 (.24)	2.94 (.26)			
	TSG	4.79 (.38)	2.73 (.31)			

CG, Control group; SG, Supplement group; TG, Training group; TSG, Training supplement group, BMI, Body mass index, FFM, Fat-free mass.

Baseline levels of adiponectin, leptin, resistin, omentin, vaspin, visfatin, apelin, RBP4, chemrin, and semaphorin3c were not different between the study groups (p>0.05), although there were interactions between group and time for these variables (p<0.05, ES range: 0.3- 0.7) *(* **Table 3** *).* Between groups comparisons analysis indicated significant differences (p<0.05) in adiponectin (ES: 0.48), leptin (ES: 0.46), resistin (ES: 0.3), omentin (ES: 0.65), vaspin (ES: 0.46), visfatin (ES: 0.62), apelin (ES: 0.42), RBP4 (ES: 0.63), chemrin (ES: 0.36), and semaphorin3c (ES: 0.5) difference among study groups in the intervention programs.

**Table 3:** Mean ( ± SD) and effect sizes of adipokines levels in the all groups.

Variables	Group	Pre-training Mean (SD)	Post-training Mean (SD)	P values (η^2^)		
				Time	G × T Interaction	Group
Adiponectin (ng/ml)	CG	10.37 (1.72)	9.2 (2.38)	<0.001 (0.55)	<0.001 (0.56)	<0.001 (0.48)
	SG	9.99 (2.34)	11.57 (1.98)			
	TG	9.14 (1.74)	13.78 (1.58)			
	TSG	10.02 (1.47)	16.29 (1.61)			
Leptin (ng/ml)	CG	6.91 (1.8)	7.28 (1.94)	<0.001 (0.6)	<0.001 (0.51)	<0.001 (0.46)
	SG	7.08 (1.92)	5.65 (1.69)			
	TG	6.66 (2.04)	4.51(1.53)			
	TSG	7.32 (2.28)	3.93 (1.92)			
Resistin (ng/ml)	CG	13.96 (2.54)	13.98 (2.77)	<0.001 (0.58)	<0.001 (0.42)	0.002 (0.3)
	SG	12.76 (2.40)	10.48 (1.66)			
	TG	13.22 (1.29)	9.85 (1.55)			
	TSG	14.93 (1.53)	9.34 (1.58)			
Omentin (ng/ml)	CG	25.26 (3.14)	24.84 (1.88)	<0.001 (0.83)	<0.001 (0.74)	<0.001 (0.65)
	SG	25.13 (3.16)	30.04 (1.46)			
	TG	25.13 (2.03)	35.52 (1.85)			
	TSG	23.64 (3.03)	38.28 (2.81)			
Vaspin (ng/ml)	CG	4.62 (.57)	4.73 (.72)	<0.001 (0.41)	<0.002 (0.3)	<0.001 (0.46)
	SG	4.05 (.73)	3.55 (0.43)			
	TG	4.30 (.95)	3.03 (0.63)			
	TSG	4.36 (0.77)	2.96 (0.49)			
Visfatin (ng/ml)	CG	21.98 (2.25)	22.33 (2.38)	<0.001 (0.44)	0.001 (0.35)	<0.001 (0.62)
	SG	21.48 (1.70)	19.33 (1.61)			
	TG	20.33 (2.13)	17.59 (1.47)			
	TSG	20.73 (2.05)	15.26 (1.81)			
Apelin (ng/ml)	CG	4.02 (.39)	3.9 (0.55)	<0.001 (0.68)	<0.001 (0.43)	<0.001 (0.42)
	SG	4.01 (.54)	3.4 (.36)			
	TG	3.92 (.42)	2.79 (.39)			
	TSG	3.93 (.51)	2.59 (.31)			
RBP4 (ng/ml)	CG	50.62 (5.94)	49.26 (2.9)	<0.001 (0.83)	<0.001 (0.7)	<0.001 (0.63)
	SG	48.81 (4.4)	40.79 (3.63)			
	TG	49.22 (3.2)	34.71 (3.22)			
	TSG	51.62 (4.05)	28.34 (4.52)			
Chemrin (ng/ml)	CG	206.66 (17.74)	208.1 (24.85)	<0.001 (0.47)	0.007 (0.26)	<0.001 (0.36)
	SG	204.74 (24.03)	173.15 (12.51)			
	TG	207.42 (15.55)	169.97 (19.49)			
	TSG	205 (24.87)	161.55 (18.14)			
Semaphorin3c (ng/ml)	CG	6.98 (1.14)	7.86 (1.05)	<0.001 (0.55)	<0.001 (0.51)	0<0.001 (0.5)
	SG	7.42 (1)	5.76 (1.07)			
	TG	7.41 (0.96)	4.26 (1.15)			
	TSG	7.32 (1.65)	3.75 (1.17)			

CG, Control group; SG, Supplement group; TG, Training group; TSG, Training supplement group.

*Post hoc* multiple comparisons analysis indicated that levels of adiponectin, leptin, resistin, omentin, vaspin, visfatin, apelin, rbp4, chemrin, and semaphorin3c were greater in participants in the TSG group followed by those in the TG and SG groups, all of which had higher levels than in the control group (p<0.05). *Post hoc* pairwise comparisons further indicated differences between all study groups (p<0.05) except when comparing leptin (p=0.25), apelin (p=0.25), vaspin (p=0.77), chemrin (p=0.31), and semaphorin3c (p=0.29) levels between TSG and TG groups. Furthermore, resistin levels did not change in pairwise comparisons between SG and TG (p=0.45) or between SG and TSG (p=0.18). Similarly, levels of chemrin were not different (p>0.05) in pairwise comparisons of either SG or TG (p=0.7) or of SG and TSG (p=0.16). Plasma levels of semaphorin3c correlated (p<0.05) with adiponectin (r= -0.63), leptin (r= 0.7), resistin (r= 0.5), omentin (r= -0.77), vaspin (r= 0.65), visfatin (r= 0.63), apelin (r= 0.6), rbp4 (r= 0.7), and chemrin (r= 0.5) levels as determined by the Pearson correlations analysis *(* **Table 4** *).* Plasma levels of semaphorin3c were significantly correlated (p<0.05) with body weight (r= 0.57), BMI (r= 0.43), FFM (r= -0.612), FAT (r= 0.768) and VO_2peak_ (r=-0.53), insulin (r= 0.756), glucose (r= 0.623), and HOMA-IR (r= 0.727) as determined by the Pearson correlations analysis *(* **Table 5** *).*

**Table 4:** Correlation analysis matrix for Adipokines.

		Adiponectin	Leptin	Resistin	Omentin1	Vaspin	Visfatin	Apelin	RBP4	Chemrin	Semaphorin
Adiponectin	r	1	-.543^**^	-.461^**^	.800^**^	-.579^**^	-.628^**^	-.634^**^	-.767^**^	-.563^**^	-.629^**^
	P		<0.001	.001	<0.001	<0.001	<0.001	<0.001	<0.001	<0.001	.005
Leptin	r	-.543^**^	1	.704^**^	-.835^**^	.626^**^	.746^**^	.641^**^	.786^**^	.566^**^	.703^**^
	P	<0.001		<0.001	<0.001	<0.001	<0.001	<0.001	<0.001	<0.001	.005
Resistin	r	-.461^**^	.704^**^	1	-.633^**^	.564^**^	.580^**^	.436^**^	.645^**^	.434^**^	.495^**^
	P	.001	<0.001		<0.001	<0.001	<0.001	.002	<0.001	.002	.005
Omentin1	r	.800^**^	-.835^**^	-.633^**^	1	-.697^**^	-.735^**^	-.715^**^	-.876^**^	-.658^**^	-.768^**^
	P	<0.001	<0.001	<0.001		<0.001	<0.001	<0.001	<0.001	<0.001	.005
Vaspin	r	-.579^**^	.626^**^	.564^**^	-.697^**^	1	.746^**^	.519^**^	.597^**^	.573^**^	.649^**^
	P	<0.001	<0.001	<0.001	<0.001		<0.001	<0.001	<0.001	<0.001	.005
Visfatin	r	-.628^**^	.746^**^	.580^**^	-.735^**^	.746^**^	1	.574^**^	.729^**^	.527^**^	.628^**^
	P	<0.001	<0.001	<0.001	<0.001	<0.001		<0.001	<0.001	<0.001	.005
Apelin	r	-.634^**^	.641^**^	.436^**^	-.715^**^	.519^**^	.574^**^	1	.739^**^	.651^**^	.604^**^
	P	<0.001	<0.001	.002	<0.001	<0.001	<0.001		<0.001	<0.001	.005
RBP4	r	-.767^**^	.786^**^	.645^**^	-.876^**^	.597^**^	.729^**^	.739^**^	1	.601^**^	.703^**^
	P	<0.001	<0.001	<0.001	<0.001	<0.001	<0.001	<0.001		<0.001	.005
Chemrin	r	-.563^**^	.566^**^	.434^**^	-.658^**^	.573^**^	.527^**^	.651^**^	.601^**^	1	.500^**^
	P	<0.001	<0.001	.002	<0.001	<0.001	<0.001	<0.001	<0.001		.005
Semaphorin	r	-.629^**^	.703^**^	.495^**^	-.768^**^	.649^**^	.628^**^	.604^**^	.703^**^	.500^**^	1
	P	<0.001	<0.001	<0.001	<0.001	<0.001	<0.001	<0.001	<0.001	<0.001	

**Correlation is significant at the 0.01 level (1-tailed).

**Table 5:** Correlation analysis semaphorin 3C with anthropomorphic markers.

		Semaphorin3c	Weight	BMI	VO2peak	FFM	FAT	Insulin	Glucose	Homa-IR
Semaphorin 3C	R	1	.569^**^	.426^**^	-.529^**^	-.612^**^	.768^**^	.756^**^	.623^**^	.727^**^
	P		<0.001	.002	<0.001	<0.001	<0.001	<0.001	<0.001	.000
Weight	R	.569^**^	1	.685^**^	-.282^**^	-.481^**^	.592^**^	.682^**^	.488^**^	.597^**^
	P	<0.001		<0.001	.032	<0.001	<0.001	<0.001	<0.001	.000
BMI	R	.426^**^	.685^**^	1	-.237	-.531^**^	.592^**^	.559^**^	.309^**^	.430^**^
	P	.002	<0.001		.061	<0.001	<0.001	<0.001	.020	.002
VO_2_peak	R	-.529^**^	-.282^**^	-.237	1	.356^**^	-.532^**^	-.466^**^	-.470^**^	-.500^**^
	P	<0.001	.032	.061		.009	<0.001	.001	.001	.000
FFM	R	-.612^**^	-.481^**^	-.531^**^	.356^**^	1	-.658^**^	-.672^**^	-.425^**^	-.562^**^
	P	<0.001	<0.001	<0.001	.009		<0.001	<0.001	.002	.000
FAT	R	.768^**^	.592^**^	.592^**^	-.532^**^	-.658^**^	1	.773^**^	.510^**^	.663^**^
	P	<0.001	<0.001	<0.001	<0.001	<0.001		<0.001	<0.001	.000
Insulin	R	.756^**^	.682^**^	.559^**^	-.466^**^	-.672^**^	.773^**^	1	.776^**^	.923^**^
	P	<0.001	<0.001	<0.001	.001	<0.001	<0.001		<0.001	.000
Glucose	R	.623^**^	.488^**^	.309^**^	-.470^**^	-.425^**^	.510^**^	.776^**^	1	.957^**^
	P	<0.001	<0.001	.020	.001	.002	<0.001	<0.001		.000
Homa	R	.727^**^	.597^**^	.430^**^	-.500^**^	-.562^**^	.663^**^	.923^**^	.957^**^	1
	P	<0.001	<0.001	.002	<0.001	<0.001	<0.001	<0.001	<0.001	

**Correlation is significant at the 0.01 level (1-tailed).

*Post hoc* multiple comparisons analysis of anthropometry, body composition, and cardio-respiratory fitness variables indicated that all pairwise comparisons between the study and control groups were different (p<0.05) except for the SG group for BMI (p=0.62), and VO_2_ peak (p=0.53). Moreover, all pairwise comparisons of SG and TG between study groups were significant (p<0.05) except for body weight (p=0.06), BMI (p=0.87), and FFM (p=0.78). Multiple comparisons of TG and TSG further indicated that there were no differences in levels of body weight (p=0.57), VO_2_ peak (p=0.78), fat mass (p=0.64), glucose (p=0.33), and HOM-IR (p=0.9). Additionally, measures of anthropometry, body composition, and cardio-respiratory fitness variables (p<0.05) were different when comparing participants in the TSG and SG groups, with no changes in FFM (p=0.14).

## 4 Discussion

The main findings of our study were that 12 weeks HIFT and spinach-derived thylakoid alone or combined improved the adipokine profiles and insulin resistance in obese men, which was greater in the TSG group, representing the synergistic effect of spinach-derived thylakoid ingestion with HIFT. Obesity is an inflammatory disease (37) that is associated with adipose tissue dysfunction and subsequent dysregulation of adipokine release (enhanced pro-inflammatory and reduced anti-inflammatory cytokine release), which in turn leads to inflammation and insulin resistance (35). Exercise training can attenuate chronic inflammation and is considered an effective anti-inflammatory strategy (38), as confirmed by our findings that exercise decreased inflammatory (leptin, resistin, vaspin, apelin, visfatin, RBP4, chemerin, semaphorin 3c) and enhanced anti-inflammatory (adiponectin and omentin) adipokine levels.

Semaphorins are extracellular signaling proteins consisting of eight classes (Sema 1-8) that play a critical role in the development and maintenance of various organs and tissues, including cardiovascular, immune, endocrine, hepatic, renal, respiratory and musculoskeletal systems (39). The class-3 semaphorin subfamily (Sema3A–3G) is involved in obesity and metabolic disorders, and utilizes neuropilins and plexins as their main binding receptors (40). Human white adipose tissue (WAT) is an important source of semaphorin 3C and greater expression is observed in the obese and metabolic syndrome, representing that semaphorin 3C plays a pathophysiological role in human WAT (6). Our study indicated significant decreases in semaphorin 3C after 12 weeks HIFT, and is in agreement with other reports where 12 weeks endurance training in healthy obese young males decreased chemerin, visfatin and semaphorin 3C levels, and with simultaneous decreases in body fat mass and insulin resistance, suggesting that changes in markers of obesity correlated with the changes in serum levels of semaphorin 3C (41).

Although exercise caused modest changes in the semaphorin 3C levels in our study, the reductions in circulation levels of semaphorin 3C we observed can be attributed to decreases in adipose tissue as a major source for secretion of this adipokines. The expression of semaphorin 3C correlates with weight change, and WAT expression decreased after weight loss through bariatric surgery, while semaphorin 3C can contribute to insulin resistance and type 2 diabetes (6). The downregulation of semaphorin 3C levels following HIFT intervention in our study led to significant decreases in insulin resistance. However, the association between semaphorin 3C and insulin resistance, and the mechanism of exercise induced decreases in semaphorin 3C levels is poorly understood unknown.

We report that 12 weeks HIFT reduced levels of leptin, resistin, vaspin, visfatin, RBP4, chemerin and increased levels of adiponectin (apelin, and omentin) levels. Leptin and resistin are inflammatory cytokines involved in the development of insulin resistance, whose levels increase with body fat mass (42). Moreover, RBP4, visfatin and chemerin increase systemic inflammation and positively correlated with insulin resistance (43). In contrast, adiponectin and omentin are anti-inflammatory adipokines that promote fatty acid oxidation, inhibit obesity and promote glucose uptake (44). Inflammation is major risk factor for various metabolic disorders including diabetes, obesity, NAFLD, atherosclerosis and cardiovascular diseases, and targeting inflammation is effective strategy for preventing and improving the inflammation-mediated disorders (45). Our findings confirmed previous reports (46) of the anti-inflammatory properties of HIFT as a new exercise training modality, and exercise training modulates adipokines levels (47, 48).

Exercise intensity modulates improvements in inflammation, as shown by our findings that HIFT improved the modulation of inflammation. Although there is little information on the effects of HIFT on adipokine profiles, but related to this is that high-intensity exercise training (HIT) caused more reductions in inflammation and endothelial dysfunction compared to low intensity exercise training (LIT) in obese adolescents (49), which can in part be ascribed to reduced adipokines secreted from adipose tissue. In this context, HIT has been shown to induce positive modulation (decrease in inflammatory adipokines such as resistin, leptin and ghrelin, and increase the anti-inflammatory cytokines such as adiponectin) of adipokine profiles in postmenopausal women with metabolic syndrome (50). Another study of obese middle-aged women reported that combined exercise training [resistance and aerobic (moderate or vigorous)] for 8 weeks lowered levels of adiponectin and leptin. Greater decreases in adiponectin levels were caused by vigorous exercise than moderate, while similar changes were recorded in leptin levels with both aerobic exercise intensities (51). The disparity related to the adiponectin changes between our finding and above-mentioned study may be related to differences in exercise intensities, exercise models, and gender differences. Although the anti-inflammatory effect of exercise training in our study can partly be attributed to losing fat mass as an important source of inflammatory mediators (52), identifying other mechanisms that may also be involved needs further investigation. However, exercise training anti-inflammatory actions can be exerted by simultaneous increases in the levels of anti-inflammatory mediators such as IL-10 (53), modulation of intracellular signaling pathways and cellular functions mediated by nitric oxide (NO) and reactive oxygen species (ROS) (54), phenotypic switching of adipose tissue macrophages from M1 to M2 and downregulation of Toll-like receptor 4 to inhibit chronic inflammation (55). Moreover, exercise training-induced improvements in the inflammatory status may also result from the secretion of skeletal muscle derived anti-inflammatory cytokines (myokine) which stimulates adipose tissue angiogenesis to increase adipose tissue blood flow (12).

Another finding of our study is that spinach-derived thylakoid supplementation alone or in combination with HIFT modulates adipokine levels in obese men, and that consuming spinach-derived thylakoid amplified the anti-inflammatory effects of HIFT function. Thylakoids can lead to positive outcomes in overweight and obesity and their related disorders by inducing satiety and suppression of hunger sensations, lowering body fat and weight, enhancing glucose homeostasis, reducing serum lipids, attenuating oxidative stress and inflammation, and reducing the absorption of dietary fat and carbohydrate (56). Thylakoids has anti-obesity activities, stimulating insulin sensitivity and improving the lipid profile through delayed glucose and lipid absorption respectively (57). By the same token, findings in an animal model of polycystic ovary syndrome (PCOS) indicated that thylakoid-treated rats showed reduced weight, attenuated HOMA-IR and insulin levels, and improved serum concentrations of lipids (58). There is, however, little information on spinach-derived thylakoid combined with exercise training on adipose tissue derived mediators, but other reports suggest that consumption of 5 g/day thylakoid-rich spinach extract along with calorie-restrictive diet in obese women improved abdominal obesity parameters, insulin resistance, and lipid profiles while decreasing the levels of inflammatory cytokines such as hs-CRP compared to the calorie-restrictive diet alone (59). However, some studies reported that spinach-derived thylakoid combined with calorie-restriction affect adipokine levels and caused significant downregulation of chemerin and upregulation of omentin in the obese women, likely due to decreases in body weight, fat mass, and BMI (22).

Thylakoids can decrease fat mass primarily by delaying fat digestion in the intestine, and is associated with increased release of the satiety hormone cholecystokinin (CCK), causing inhibition of gastric emptying and lowering the distension of the stomach, and stimulating the release of satiety molecules such as serotonin (60). In addition, animal studies indicated that 100 days of feeding mice with thylakoids increased pancreatic lipase activity and serum CCK levels and decreased serum levels of PYY and leptin (61).

Our study shows that HIFT and thylakoids improve lipid profiles and increases VO_2peak_. One reason for the lower levels of plasma cholesterol is the reduction of plasma insulin. A decrease in plasma insulin activates lipolysis of adipose tissue, so increasing free fatty acid levels in the plasma and liver. At the same time as insulin decreases, levels of glucagon increase, and both hormones stimulate ketogenesis and cause changes in cholesterol precursors during exercise (62, 63). In terms of the mechanism involved in the process of reducing LDL-C, it is known that performing sports activities increases the activity of lipoprotein lipase (LPL) and decreases hepatic triglyceride lipase. Increasing the activity of LPL stimulates the catabolism of lipoproteins which are rich in triglycerides, thus causing levels of LDL-C to decrease with physical activity (64). Improvements of triglyceride changes can also be attributed to the response of LPL to exercise training. On the other hand, regular exercise reduces and inhibits the activity of liver lipase (65), causing a reduced production of VLDL-C and LDL-C. A likely cause of the increases in HDL-C following exercise training is the role of exercise in stimulating factors involved in the formation and transformation of HDL-C, such as LPL and lecithin–cholesterol acyltransferase (LCAT), phospholipid transfer protein (PLTP) and ATP-binding cassette (ABC) protein (66). One reason for reduced insulin resistance in our study is the role of exercise in modulating muscle signaling in fasting conditions through inhibiting the AKT/PKB pathway (67). The AKT/PKB signaling pathway represents a primary mechanism by which insulin regulates glucose transport in skeletal muscle. Therefore, a reduction in the AKT/PKB signaling pathway in skeletal muscle by exercise could mediate improvements in insulin resistance (68).

Despite these findings, there is limited information on the mechanisms of spinach-derived thylakoid combined with exercise training in improving health outcomes.

### 4.1 Study limitations

Our study has several limitations. Firstly, we did not identify the mechanisms by which bioactive components of spinach-derived thylakoid can improve adipokines levels. Second, our study cannot be generalized as females were not included in patient recruitment. The third is that we did not measure levels of chlorophyll and other thylakoid components.

## 5 Conclusions

Our study provides novel information on the beneficial effects of a combination of spinach-derived thylakoid supplementation with HIFT in the management of adipokines in obese males. Our data suggests that nondrug strategies such as spinach- derived thylakoid supplementation with HIFT can have protective effects on adipokines, glucose homeostasis parameters (insulin, glucose and insulin resistance); and body composition variables (weight, FM and FFM).

## Data availability statement

The raw data supporting the conclusions of this article will be made available by the authors, without undue reservation.

## Ethics statement

The studies involving human participants were reviewed and approved by The Research and Ethics Committee of the Islamic Azad University approved all procedures of this study (Ethics code: IR-IAU1400-46). All procedures were performed according to the latest revision of the Declaration of Helsinki. The patients/participants provided their written informed consent to participate in this study.

## Author contributions

All authors contributed equally to data collection. They read and approved the submission of the final version of the manuscript.
